# Opportunities and Challenges of Telehealth in Remote Communities: Case Study of the Yukon Telehealth System

**DOI:** 10.2196/11353

**Published:** 2019-11-01

**Authors:** Emily Seto, Dallas Smith, Matt Jacques, Plinio Pelegrini Morita

**Affiliations:** 1 Institute of Health Policy, Management, and Evaluation University of Toronto Toronto, ON Canada; 2 Centre for Global eHealth Innovation Techna Institute University Health Network Toronto, ON Canada; 3 Health System Improvement and Transformation Department of Health and Social Services Government of Yukon Whitehorse, YT Canada; 4 Government Internal Audit Services Executive Council Office Government of Yukon Whitehorse, YT Canada; 5 School of Public Health and Health Systems Faculty of Applied Health Sciences University of Waterloo Waterloo, ON Canada

**Keywords:** health care systems, telemedicine, remote consultation, Yukon territory, telehealth, program evaluation, medical informatics

## Abstract

**Background:**

Telehealth has been shown to improve access to health care and to reduce costs to the patient and health care system, especially for patients living in rural settings. However, unique challenges arise when implementing telehealth in remote communities.

**Objective:**

The study aimed to explore the current use, challenges, and opportunities of the Yukon Telehealth System. The lessons learned from this study were used to determine important factors to consider when attempting to advance and expand telehealth programs in remote communities.

**Methods:**

A mixed methods approach was used to evaluate the Yukon Telehealth System and to determine possible future advances. Quantitative data were obtained through usage logs. Web-based questionnaires were administered to nurses in each of the 14 Yukon community health centers outside of Whitehorse and patients who had used telehealth. Qualitative data included focus groups and semistructured interviews with 36 telehealth stakeholders.

**Results:**

Since 2008, there has been a consistent number of telehealth sessions of about 1000 per year, with clinical care as the main use (69.06% [759/1099] of all sessions in 2015). From the questionnaire (11 community nurses and 10 patients) and the interview data, there was a consensus among the clinicians and patients that the system provided timely access and cost savings from reduced travel. However, they believed that it was underutilized, and the equipment was outdated. The following 4 factors were identified, which should be considered when trying to advance and expand a telehealth program: (1) patient and clinician buy-in: past telehealth experiences (eg, negative clinician experiences with outdated technology) should be considered when advancing the system. Expansion of services in orthopedics, dermatology, and psychiatry were found to be particularly feasible and beneficial in Yukon; (2) workflow: the use and scheduling of telehealth should be streamlined and automated as much as possible to reduce dependencies on the single Yukon telehealth coordinator; (3) access to telehealth technology: clinicians and patients should have easy access to up-to-date telehealth technology. The use of consumer products, such as mobile technology, should be leveraged as appropriate; and (4) infrastructure: the required human resources and technology need to be established when expanding and advancing telehealth.

**Conclusions:**

While clinicians and patients had generally positive perceptions of the Yukon Telehealth System, there was consensus that it was underutilized. Many opportunities exist to expand the types of telehealth services and the number of telehealth sessions, including the expansion of services in several new specialty areas, updating telehealth equipment to streamline workflows and increase convenience and uptake, and integrating novel technologies. The identified barriers and recommendations from this evaluation can be applied to the development and expansion of telehealth in other remote communities to realize telehealth’s potential for providing efficient, safe, convenient, and cost-effective care delivery.

## Introduction

### Background

Telehealth services have become an integral part of health care in many jurisdictions within Canada and internationally [1-6]. Studies have found that telehealth can improve access to health services, improve health outcomes, reduce costs, and increase educational opportunities [7]. In particular, access to telehealth services for citizens in remote or underserved areas enables access to health care and programs that may otherwise be unavailable and reduces wait time, costs to the health care system, and personal expenses related to the patient’s travel to reach health care services in urban centers [8,9]. Previous studies have found that telehealth increased access to health services, enhanced educational opportunities and social support, and improved health outcomes, quality of care, quality of life, and cost-effectiveness [7].

Telehealth provides substantial opportunities to improve health outcomes and service delivery, while reducing costs in regions such as Yukon, Canada, that have remote communities, centralization of health care services within a few cities, and significant reliance on out-of-territory clinical specialists [10,11]. However, the same remote nature of Yukon’s communities outside of Whitehorse, with populations between 50 and 2500, provides some of the main challenges for the use and wide-scale deployment of telehealth technology.

The existing Yukon Telehealth System is used to serve the 38,450 inhabitants of Yukon (accurate as of 2017) [12], and is used for clinical care, clinician education, and administration. It comprises mobile telehealth units that are mainly used for clinical care and desktop telehealth software that is used for educational and administrative purposes. Each of the 14 community health centers has a single telehealth unit, and additional telehealth units are located in major centers such as Whitehorse. The system is managed by a single telehealth coordinator. Her duties include scheduling, initiating the scheduled telehealth sessions, technical support, and general oversight of the Yukon Telehealth System. Patients travel to one of the community health centers or other sites with telehealth units to participate in the scheduled telehealth sessions.

### Objectives

The objective of this evaluation was to understand the current use, challenges, and opportunities of the current Yukon Department of Health and Social Services Telehealth System. The evaluation was initiated as part of the improvement initiatives focused on Mental Health, Addictions, and Chronic Conditions Support Program areas and aimed at providing a better understanding of the current state of the system that will enable the Yukon government to explore options to expand and advance the current system. The opportunities and barriers identified, as well as the provided recommendations, can be used to help guide other telehealth programs for remote communities to increase adoption and promote expansion of telehealth services.

## Methods

### Study Design Overview

A mixed methods approach was used to evaluate the Yukon Telehealth System and to determine the possible future advances to their existing infrastructure and services, combining interviews, focus groups, and site visits, with additional quantitative metrics using questionnaires. The Clinical Adoption Framework, which includes macroconstructs, mesoconstructs, and microconstructs that can influence the implementation and successful use of health care technologies, was used to guide the evaluation [13]. Data were collected between April 17, 2016, and August 2, 2016. The evaluation was aimed at answering the following 2 research questions:

1. What are the perceptions and use of the current Yukon Telehealth System?
2. What are the challenges and opportunities to improving the Yukon Telehealth System?

### Quantitative Data

To determine the number and types of telehealth sessions that have occurred, usage logs were obtained and analyzed. Information on each telehealth session arranged by the telehealth coordinator was regularly logged in a comprehensive Excel (Microsoft Corporation) log file. Every record entered on the log file was time stamped and tagged using structured labels for the (1) type of call (administrative, educational, and clinical care), (2) location of the call initiation, (3) call duration, (4) location of external members (external to the territory), and (5) Yukon sites involved in the call. Other indicators such as more granular types of calls were logged using unstructured labels. The data were structured in a tabular format and logged using binary variables for each indicator described above.

These data were analyzed to determine usage patterns over the years, including the purposes of the telehealth sessions, number of sessions in different categories, and the location of the sessions. These usage logs were validated by comparing them with the data generated by the telehealth platform (Cisco Telepresence Management Suite) and with the telehealth billing data.

Community nurses and patients were asked to complete questionnaires regarding their use and perceptions of the telehealth system. A community nurse from each of the 14 Yukon communities, outside of Whitehorse with a telehealth unit, was sent an email with a link asking them to complete the Web-based questionnaire. During a regular visit to the community health center, patients who had previously used telehealth services were asked by the community nurses and clerks if they would be willing to voluntarily complete either a paper copy or Web-based version of a questionnaire. Completed paper copies of the questionnaires were mailed back to the evaluation team using a prestamped and addressed envelope. Both community nurses and patient questionnaires included a section enabling free-text comments. The questionnaire data were analyzed using descriptive statistics. Further statistical analysis was not possible owing to the small sample size of the responses to the questionnaires.

### Qualitative Data

Individual semistructured interviews were conducted with stakeholders and users and 3 focus groups to gain an understanding of the current use, satisfaction, and perceived challenges and opportunities of the Yukon Telehealth System. Face-to-face interviews were conducted during site visits to Whitehorse, Carcross, and Dawson City. In total, 23 stakeholders participated in the face-to-face focus groups/individual interviews. The interview guide was developed to explore each relevant construct of the Clinical Adoption Framework [13].

In addition to the onsite interviews and focus groups, telephone interviews were conducted with (1) 4 community nurses, 1 each from Dawson City (population 1860), Watson Lake (population 1550), Beaver Creek (population 100), and Faro (population 400); (2) 9 physicians who provide services in Yukon (dermatologists, orthopedic surgeons, ophthalmologists, psychiatrists, and the chief of medical staff); (3) the manager of telehealth core services in British Columbia; and (4) 2 members of the Ontario Telemedicine Network. The outpatient services office manager at Whitehorse General Hospital referred the specialists for the interview as they were deemed as physicians who may be interested in using the telehealth services or who were already providing services in Yukon. The specialists were all out-of-territory health care providers as they flew periodically into Yukon to provide clinical services but were not residents of Yukon. In addition, a face-to-face interview was conducted with a previous manager of the telehealth services in Ontario. In total, 40 stakeholders of the Yukon Telehealth System and telehealth experts were consulted during this evaluation. A summary of the stakeholders interviewed is presented in Table 1.

**Table 1:** Study participants organized by methods and by groups used for data collection.

Method, roles or groups		Sample size, n
**Face-to-face focus groups and interviews**		
	Yukon telehealth users	23 (community nurses, physicians, and administrators)
	External telehealth specialists	1
**Phone interviews**		
	Yukon specialists (from other provinces)	9 (dermatologists, orthopedic surgeons, ophthalmologists, psychiatrists, and the chief of medical staff)
	Community nurses	4 (Dawson City, Watson Lake, Beaver Creek, and Faro)
	Managers	1
	External telehealth specialists	2

Furthermore, 2 researchers (ES and PPM) were present for all interviews and focus groups. Extensive notes were taken by the researcher who was not the main facilitator of the interview/focus group. All interviews and focus group sessions were also audio recorded but were not transcribed. A thematic analysis [14] was conducted, whereby the 2 researchers discussed the findings from each of the interviews/focus groups, immediately after each session, with the aid of the notes that were taken to determine emerging themes. The themes were added to the list of generated themes after each session. As the interview guide used for each of the interviews/focus groups followed the constructs of the Clinical Adoption Framework, the qualitative data largely followed the same format which facilitated thematic analysis. Any discrepancies in the interpretation of the interviews/focus groups were discussed until consensus was reached. After all the interviews/focus groups were completed, the 2 researchers convened to finalize the derived themes. The recordings were reviewed as necessary to refresh the memory of the researchers and to extract relevant quotes. Finally, the themes were presented and discussed with 2 other members of the study team (DS and MJ) who worked in the Health and Social Services of the Yukon government.

The quantitative and qualitative findings were triangulated through discussions among all authors to provide a comprehensive understanding of the current state of the system and the potential for an improved future Yukon Telehealth System.

## Results

### Current Use of the Yukon Telehealth System

The Yukon Telehealth System was first deployed in 2006 and has had no substantial upgrades since that time. Each of the 14 community health centers (Beaver Creek, Carmacks, Dawson City, Destruction Bay, Haines Junction, Mayo, Pelly Crossing, Old Crow, Watson Lake, Teslin, Carcross, Ross River, Faro, and Whitehorse sites) has a single mobile telehealth unit; additional telehealth units are located in major centers such as Whitehorse and Dawson City. Other stationary telehealth units are mounted on the walls of boardrooms and meeting rooms in major centers (Whitehorse, Dawson City, etc). A complementary telehealth desktop application was installed on the computers used by the nurses in charge in the communities to attend meetings remotely and for peer consultations, while they were at their workstations. The desktop application was not used for clinical care with patients.

**Figure 1:**
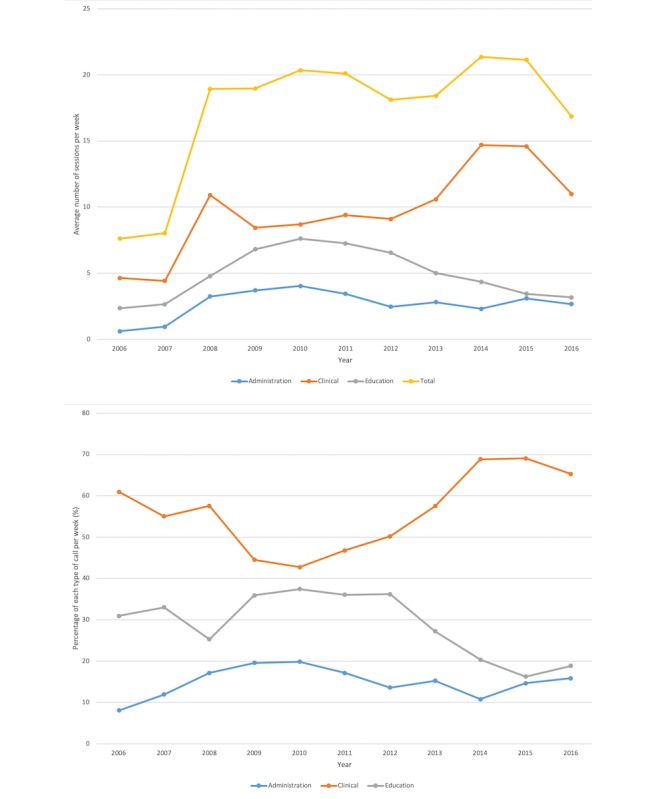
Telehealth usage by the number of sessions per week and the percentage of calls per week, collected from the log file generated by the telehealth coordinator and organized by the different types of calls.

The Yukon Telehealth System was being used for 3 major purposes: (1) clinical care, (2) clinician education, and (3) administration. There has been a consistent number of telehealth sessions of approximately 1000 sessions per year since 2008, with the main use of the telehealth system being clinical care. The total number of calls for each year for the 3 categories (administration, educational, and clinical care), along with the total number of billed consultations in the Yukon for each year are presented in Table 2. The same data are presented in a graphical form in Figure 1. On an average, 4.61% (5345/115,867) of the specialist consultations were delivered through telehealth.

The specific use of the telehealth system was recorded by the telehealth coordinator on an Excel log file. A total of 676 unique labels existed on the dataset, with some labels only logging a couple of calls over the years. The top 10 specific reasons for the telehealth sessions are displayed in Table 3. The information presented in Table 3 provides the number of calls for each type of telehealth session, in addition to the corresponding percentage of yearly calls associated with that specific use. The top 10 uses of the telehealth platform shown in the table account for 40% to 50% of all telehealth calls for each year. Owing to the complexity of the log file and the lack of a systematic labeling structure, further analyses were not possible.

**Table 2:** The total number of calls per year for 3 different purposes (administrative, educational, and clinical care).

Year	Telehealth calls, n			Billed Consultations, n (% via telehealth)
	Administration	Clinical	Education	
2006	24	181	92	8975 (2.02)
2007	50	230	138	9681 (2.38)
2008	169	567	249	9453 (6.00)
2009	193	439	354	9120 (4.81)
2010	210	452	396	10,246 (4.41)
2011	179	489	377	10,619 (4.60)
2012	128	473	341	10,499 (4.51)
2013	146	551	261	11,595 (4.75)
2014	120	764	226	12,152 (6.29)
2015	161	759	179	11,708 (6.48)
2016	107	440	127	11,819 (3.72)
Total	1487	5345	2740	115,867 (4.161)

**Table 3:** Specific use of the telehealth platform (top 10). Numbers presented in the table indicate the total number of sessions logged with that specific label and the percentage of that type of session for each year.

Reason for session		Year										
		2006	2007	2008	2009	2010	2011	2012	2013	2014	2015	2016
**Clinical care,** **n (%)**												
	Diabetic education follow-up with patients	20 (7)	59 (13)	144 (15)	105 (11)	92 (9)	68 (7)	71 (8)	74 (8)	59 (5)	61 (6)	26 (4)
	AA^a^ meetings	0 (0)	1 (0)	22 (2)	41 (4)	46 (4)	45 (4)	51 (5)	46 (5)	50 (5)	51 (5)	35 (5)
	Counseling (mental health patient/professional)	1 (0)	6 (1)	72 (7)	103 (10)	35 (3)	42 (4)	23 (2)	21 (2)	21 (2)	7 (1)	9 (1)
	Occupational stress injury	0 (0)	0 (0)	0 (0)	0 (0)	0 (0)	16 (2)	1 (0)	53 (6)	106 (10)	97 (9)	55 (8)
	Doctor’s appointment (mental health)	0 (0)	0 (0)	0 (0)	0 (0)	0 (0)	0 (0)	0 (0)	61 (6)	88 (8)	80 (7)	38 (6)
	Interview (general interviews)	8 (3)	11 (2)	27 (3)	48 (5)	45 (4)	48 (5)	30 (3)	23 (2)	13 (1)	6 (1)	0 (0)
	Cancer patient appointment	0 (0)	0 (0)	0 (0)	0 (0)	0 (0)	6 (1)	21 (2)	49 (5)	65 (6)	74 (7)	43 (6)
	Surgeon’s clinic (virtual surgeon’s appointment with patients in community)	0 (0)	0 (0)	0 (0)	0 (0)	0 (0)	0 (0)	0 (0)	0 (0)	90 (8)	76 (7)	62 (9)
**Educational,** **n (%)**												
	Rural mental health supervision	0 (0)	2 (0)	40 (4)	20 (2)	57 (5)	29 (3)	14 (1)	18 (2)	22 (2)	17 (2)	9 (1)
**Administrative,** **n (%)**												
	General meetings	16 (5)	35 (8)	91 (9)	83 (8)	107 (10)	82 (8)	52 (6)	76 (8)	63 (6)	85 (8)	34 (5)

^a^AA: Alcoholics Anonymous.

### Perceptions of Clinicians Residing in Yukon

Clinicians residing in Yukon were generally satisfied with the telehealth system. In particular, users of the telehealth system cited the quality of the telehealth coordinator’s work and commitment to the operation of the platform as the key factors in their satisfaction with the system. The clinicians residing in Yukon who were interviewed believed that telehealth had several benefits to the health care system, clinicians, and patients, including the following:

- Saving patients’ time and money by reducing travel to urban centers and hospitals (ie, patients would not have to take as much time off from work to attend consultations).
- Saving government funds by not having to pay for the patients’ travel expenses to go to urban centers for consultations that could have been delivered through telehealth.
- Improving the patients’ quality of care by providing more timely and convenient access to clinical care.
- Preventing isolation as it was reassuring to clients to see the clinician’s face when isolated in remote communities. Prevention of isolation was also cited as a benefit of using telehealth to connect social worker staff as isolation is one of the main contributors to burning out.
- Preventing unnecessary medevac cases and, therefore, reducing the need for nurses to leave the community health centers (nurses travel with patients to hospital in medevac cases).
- Multiple family members and care providers can be included in the same telehealth session, improving the overall awareness of the patients’ conditions and care plans.
- Enabling community members to participate in programs not offered in their local community, such as Alcoholics Anonymous (AA) meetings.

**Figure 2:**
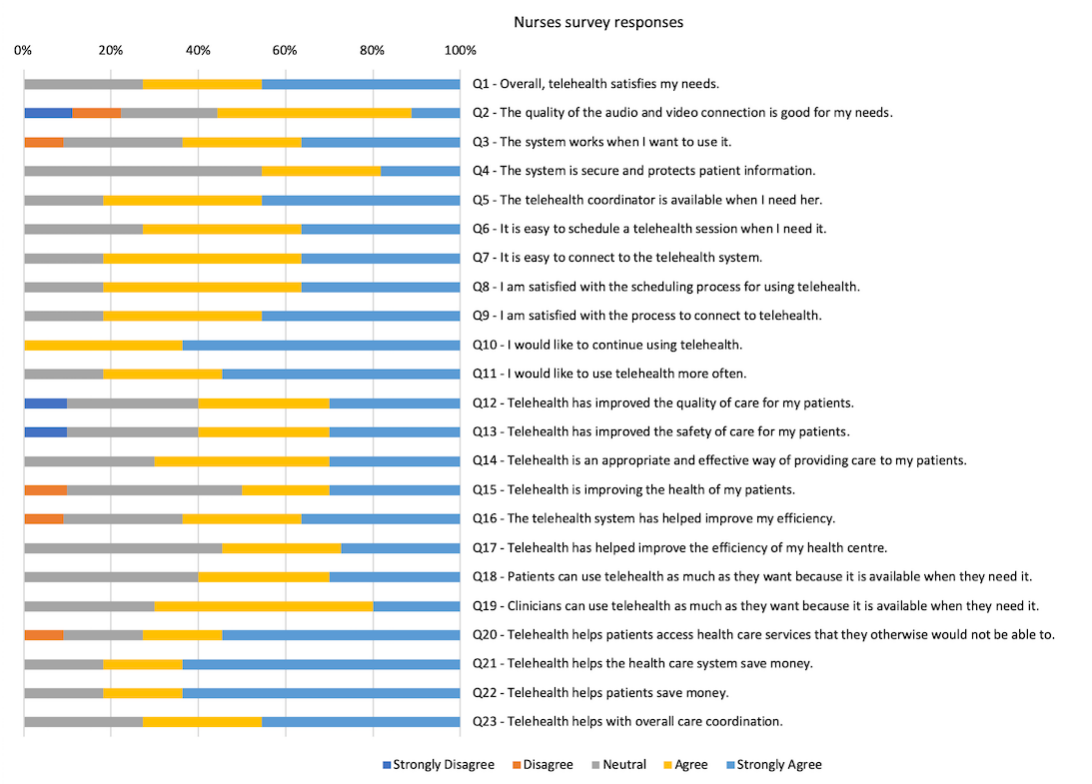
Results from the community nurse questionnaire. A total of 11 out of 14 nurses completed the questionnaire.

The questionnaire responses provided by the community nurses (11 nurses completed a questionnaire out of the 14 nurses that were invited to participate) supported the information gathered at the interviews in that the responses were generally neutral to satisfactory with the telehealth system (see Figure 2). However, the interviewed clinicians identified several limitations of the telehealth system, including the following:

- Suboptimal, outdated, and complex technology, combined with complex workflows to access and use the technology, resulting in the underutilization of the infrastructure for clinical purposes. A clinician commented, “I hope we could use it more, but the technology is not there yet.” Another clinician stated, “The system seems old and clunky.”
- Dependency on a single telehealth coordinator resulted in the loss of service quality and associated system knowledge when that individual was unavailable (vacation, sick leave, retirement, etc), even considering the existence of a backup person. One of the interviewed clinicians stated, “If we didn’t have (the telehealth coordinator), we would not have a program.”
- Nonexclusive telehealth rooms (rooms shared for meetings/boardrooms) resulted in telehealth units not being available when needed, in addition to not providing a comfortable and conducive environment for the patient.
- Inadequate training of users of the telehealth technology led to an overreliance on the telehealth coordinator.
- Not all sessions or specialties are suitable for telehealth, as some may still require a face-to-face meeting for a comprehensive assessment of the patients’ health.
- Limitations in the availability of telehealth units, lack of access of telehealth on their own desktops, and inconvenient setup process resulted in out-of-territory physicians spending more time to see a telehealth patient compared with an in-person visit by a patient to their office.
- Complexity and being unaware of the territories’ billing process limited the willingness of the out-of-territory providers to use telehealth.

### Out-of-Territory Specialists’ Perceptions

The out-of-territory providers that were interviewed fell under 1 of the 4 specialties: dermatology, psychiatry, orthopedics, and ophthalmology. Each of the 4 specialties had different perspectives on the potential benefits of telehealth and different ideas on how to expand the use of the platform, as outlined below:

- Dermatology: The out-of-territory providers mentioned that dermatology was an area that could greatly benefit from the use of telehealth in terms of store-and-forward and live consultations in conjunction with inspection cameras.
- Psychiatry: Telehealth could be extremely useful to connect to Yukon clients between the psychologist’s visits to Whitehorse, and it could also prevent clients from driving long distances to come for a consultation in Whitehorse. The benefits of telehealth for group sessions, such as AA meetings, were also highlighted.
- Orthopedics: Telehealth was viewed as a potential way for orthopedic surgeons to assess more Yukon citizens, to triage patients (ie, prioritize urgent cases) before a surgical consultation, and for postsurgical follow-ups. The presence of a physiotherapist with the client would enable the surgeon to assess whether the client should be scheduled for a face-to-face consultation in Whitehorse.
- Ophthalmology: A more systematic and secure method of sending ophthalmology images may be of use (store-and-forward), particularly for any future screening programs for diabetic clients. It was also believed that nurses trained to dilate the pupil could take the images locally and send the images along with relevant client information (eg, blood pressure, hemoglobin A1c, and blood glucose levels) for review. It was also suggested that the camera on mobile phones, particularly with an adapter lens, would be sufficient to take images for screening sessions that were noncritical. However, telehealth sessions between clients and ophthalmologists did not seem to be of benefit.

Although most specialists stated that they perceived the potential benefit of telehealth, 2 themes of barriers specific to the specialists emerged from the interviews. The first barrier was that the specialists were already too busy and there was a lack of incentives to use telehealth. The specialists commented that they already had full schedules with wait lists for their clients in their home location. Therefore, there were not a lot of incentives (financial and otherwise) to start seeing more Yukon clients with telehealth. An additional complication was revealed by one orthopedic surgeon who discussed that he was only allowed to perform a certain number of surgeries on joints per year. The number of joint surgeries he performs in Yukon would get deducted from his quota of clients in his home province. The second barrier was with regard to the challenges with scheduling telehealth consultations. Many specialists commented on the difficulty of scheduling telehealth sessions between face-to-face consultations because of the timing (ie, clients being late or not showing up for telehealth sessions, time required to connect via telehealth, normal backlog of in-person clients, etc).

### Patients’ Perceptions

**Figure 3:**
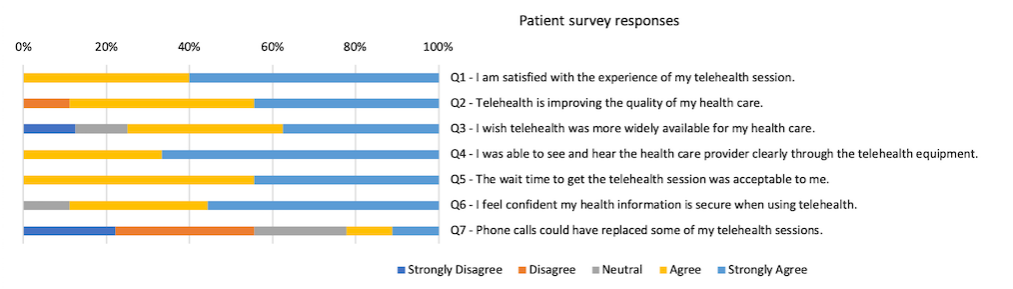
Results from the patient questionnaire. A total of 10 patients completed the questionnaire used in this study.

The patients’ perspective was collected through patient questionnaires and indirect information provided by the clinicians. A total of 10 patients completed the questionnaire. The results from the patient survey are provided in Figure 3. Collecting additional data through patient interviews was considered infeasible by the evaluation team owing to the infrequent use of telehealth in each community and the remoteness of the communities likely leading to challenges in recruitment. In addition, the small population size of the remote communities could present challenges in assuring patient confidentiality during the interviews.

Patients perceived telehealth to be extremely important to the quality of their care and wanted it to be more widely available. In general, the patients were satisfied with their telehealth experiences, including the quality of the sessions, security, and wait times. Some quotes from patients who provided comments on the questionnaire, support the perceived benefits from telehealth:

I only have used telehealth for an AA meeting, but it is extremely significant to my recovery.

Patient

Excellent service for the communities.

Patient

I appreciate the convenience of going to the Health Centre versus driving an hour to Whitehorse for doctors’ appointments.

Patient

The interviewed clinicians believed that the clients would want to use telehealth more often but were not aware that it was available. Instead, patients would often ask if it was possible to use Skype (Microsoft Corporation). Clinicians believed that many patients would find the reduction of travel to be the main benefit of telehealth, especially as many lived in remote areas and the weather in Yukon can be a barrier to traveling. By not having to travel to their health care appointments, clients would not need to take as much time away from work, which can be especially important depending on their jobs (eg, storekeepers and farmers). However, several clinicians mentioned that some clients would want to continue traveling to Whitehorse for their appointments as they are reimbursed for their travel and hotel costs, and that it is an opportunity for them to get chores (eg, shopping and visiting friends and family) accomplished while they are in Whitehorse for their consultations.

## Discussion

### Principal Findings

This evaluation explored the current use and perceptions of the Yukon Telehealth System, as well as the opportunities and challenges to improving the system. Although the clinicians’ and patients’ experiences with telehealth have been generally positive, there was a consensus that telehealth services were underutilized, which is in alignment with other studies in the field [11]. This qualitative finding was supported by the observed plateau in the number of telehealth sessions since 2008, which can be seen in Figure 1. Several factors could potentially explain the occurrence of such a plateau, which include (1) the system reaching the maximum capacity to handle calls, (2) limited capacity of the telehealth staff to handle more calls, (3) limited access to telehealth-equipped locations to initiate and receive calls, (4) limited interest by physicians and practitioners to use the telehealth system, or (5) limited awareness about the telehealth services and capabilities. Such results indicate that the limitations are likely at the system level, providing several opportunities for improvement as discussed below. However, since 2012 there has been an increase in the use of telehealth for clinical purposes and a decrease in the use for educational purposes. There was no direct evidence on the reasons for this shift in the use of the Yukon Telehealth System, but it could be because of an increase in the desire to use telehealth for clinical purposes owing to an improved perception of its clinical benefits; however, this resulted in less availability of the system for educational purposes.

The main contribution of this case study is the determination of 4 overarching factors related to sustainability, quality improvement, and scalability that should be considered when attempting to advance and expand telehealth systems in remote communities such as in Yukon. These factors can be easily overlooked or not properly addressed, which can lead to the stagnation and underuse of telehealth programs, as was the situation in this case study. This case study provides specific examples of how these factors have impacted the growth and adoption of telehealth in a remote setting. These factors are generally aligned with the findings from other telehealth studies in similar environments [11,15]. Each of these 4 factors is discussed below with recommendations on how to address the identified issues. See Table 4 for a summary of the factors and recommendations.

**Table 4:** Overarching factors and recommendations for the expansion of telehealth systems.

Factor	Implications if not addressed	Recommendations
Patient and clinician buy-in	Underutilization of the telehealth program owing to lack of interest, resulting in wasted resources.	Leverage existing patient and clinician buy-in to focus on the specific applications of telehealth. Consider population-specific social drivers and goals. Capitalize on the existing clinical interest to identify clinical champions.
Workflow	Telehealth sessions may take more time than face-to-face consultations, resulting in clinician frustration and decision to stop using telehealth. Poor patient satisfaction with telehealth owing to scheduling delays.	Ensure that scheduling and initiation to telehealth services are quick and easy (ideally directly between the provider and patient).
Access to telehealth technology	Clinicians spending time relocating to another room or not having a suitable time slot for the telehealth session, leading to frustration and decision to stop using telehealth. Telehealth being inaccessible to patients as they cannot physically get to the location of the telehealth site.	Enable clinicians to provide telehealth services from their own offices with desktop solutions instead of relocating to other rooms. In case there is a separate telehealth room, ensure that it is accessible at all times with a priority for telehealth use. Provide options for patients to access telehealth from their own homes.
Infrastructure	Lack of appropriate human resources and technological infrastructure can result in telehealth services being unavailable if staff are away (eg, become sick) or the technology has a point of failure.	Ensure redundancy of telehealth staff (ie, do not solely rely on a single telehealth coordinator). Ensure that the telehealth coordinator has the time and resources for quality improvement initiatives. Develop detailed training and maintenance plans. Consider multiple points of access to telehealth services for patients, such as through consumer mobile devices.

### Patient and Clinician Buy-In

The overall perception of patients and clinicians, both from interviews and questionnaires, indicated perceived value to using telehealth in Yukon, which include reduced need to mobilize patients while creating opportunities to connect patients and physicians on a more regular basis. The desire to expand telehealth services was voiced by both clinicians and patients.

However, program evaluations should also consider population-specific social drivers and goals. For example, some patients may prefer to travel for clinical visits as it is an opportunity for paid travel to Whitehorse to perform other errands in the city. Other challenges also include past negative experiences with telehealth (eg, past difficulty to use or access telehealth services, connectivity issues, and scheduling conflicts), which directly influence their willingness to use the system in the future.

A major opportunity to expand the use of telehealth services is through clinical champions who have indicated particular interest in using telehealth, as demonstrated by Wade et al [16]. Our interviews found strong interest from champions in Yukon in the areas of orthopedics, dermatology, and psychiatry, highlighting a relevance for both physical (orthopedics and dermatology) and social (psychiatry) interactions with patients. These specialties have had recent significant developments in telehealth [17-20], which indicate that expansion in these areas in Yukon may be particularly beneficial. A study of future developments of telehealth in Western Australia found that their top 4 most needed telehealth services were wound care, emergency, psychiatry, and ophthalmology [21], which somewhat differed from our case study. This points to the potential differences in targeting applications of telehealth for implementation, depending on the current opportunities and clinical buy-in of the jurisdiction.

### Workflow

One of the barriers identified by the clinicians was the current cumbersome workflow related to scheduling calls. Telehealth users expect the scheduling process to be seamless and easy, similar to scheduling calls via other current communication channels, such as through the phone or Skype. The availability of consumer technology in the market that provides patients with a better experience is a significant driver for improvements in other services [22]. In the case of the Yukon Telehealth System, clinicians expect to be able to schedule a call directly with the patient, without having to go through telehealth coordinators. Anecdotal information collected during this study from clinicians that currently use telehealth in Yukon also indicate that patients share the same feeling and would benefit from the opportunity to take calls from the convenience of their own home, as explored by DelliFraine and Dansky [23] and Bensink et al [24].

This evaluation found that clinicians perceived that owing to complexities in the workflow and the limitations of the telehealth system, some telehealth calls require more time than a regular in-person consultation session, which is consistent with the findings in the literature [11]. To deliver a positive user experience to clinicians, an ideal telehealth platform should enable clinicians to initiate and receive calls from their own office and connect directly to the patient, minimizing uncertainties introduced by a cumbersome workflow and allowing users to initiate their own sessions. Although specific schedules for telehealth were identified as a condition for telehealth implementation in previous studies [11], our study found that establishing an easy scheduling procedure was a key factor to increase adoption.

For a telehealth system to be successful and support expansion, an improved and direct scheduling system should be implemented to deliver a more streamlined workflow. This scheduling system should allow direct and automatic scheduling by patients and clinicians to schedule their own sessions with a telehealth coordinator who can provide oversight for conflicts and prioritization.

### Access to Telehealth Technology

In line with the workflow difficulties, getting physical access to telehealth units (rooms too far from their main workplace or rooms inaccessible at the time of the call) was also described as a significant issue. The Yukon telehealth equipment was usually in rooms that were used for multiple activities, and clinicians commented that on several instances, they were not able to use the technology as the space was booked for meetings or face-to-face consultations. Consequently, the clinicians’ daily activities were disrupted if they had to relocate for telehealth sessions, which was compounded by cases in which patients did not show up for their telehealth session.

The solutions to some of these recurring issues are complementary to those identified in the workflow section, where the telehealth systems should provide expanded desktop-based telehealth services that would enable clinicians to make and receive calls directly from their offices and provide dedicated telehealth space for the telehealth units, when more specialized equipment is necessary (cameras, vitals sensors, etc). Numerous technologies in the market can provide secure, desktop-based telehealth services such as Jabber (Cisco Systems) and Skype for Business, among others. The use of desktop-based technology would enable physicians to schedule multiple sequential sessions directly from their office, which would minimize the impact on their face-to-face consultations and workload [25].

Patients have also shown a strong interest in being able to minimize the number of visits to their local community health center to receive telehealth, as a trip could be a hazardous endeavor in winter months in Northern Canada. The ability to connect via telehealth directly from home using their own home devices, such as mobile phones and tablets, would provide a significant improved experience for these patients and potentially reduce issues related to mobilizing sick patients to community health centers. Similar home-based services have been widely presented by other authors in the field, showcasing the widespread benefit of enabling patients to attend their visits from the comfort of their own home [26-31].

### Infrastructure

The fourth factor relates to the infrastructure (human resources and technology) necessary to operate a telehealth system. The evaluation identified an understaffed telehealth team, with overdependence on a single telehealth coordinator. A telehealth assistant or a second coordinator could be employed to add redundancy for the current telehealth coordinator, improving the overall service quality for telehealth users. The automation of currently manual procedures (eg, scheduling and telehealth session initiation) could free up time for the telehealth coordinator to conduct continuous quality improvement initiatives that would improve the overall experience of telehealth users.

Auxiliary services that are often not considered when implementing a telehealth service include training and maintenance staff to support the telehealth units that are in remote communities. Owing to a high turnover rate of staff in remote locations, a training plan is particularly essential. Training and maintenance are especially important with regard to the Yukon Telehealth System because of its outdated equipment, which has components that can no longer be procured if broken.

The ideal shift in technology should include a combination of more modern telehealth units, telehealth desktop clients, and consumer mobile devices to be used based on the application and needs of the users. The use of consumer mobile devices for telehealth is a relatively new concept and a potential area of future research. An integrated, multiplatform system would deliver a much better experience to patients and clinicians, potentially increasing the usage of telehealth in Yukon and expanding the accessibility of the telehealth service to underserved groups. Such an integrated solution can potentially include modules for scheduling sessions, initiation of sessions, call tracking, and quality improvement.

### Limitations

The limitations of this evaluation include an underrepresentation of the patient perspective owing to a low patient questionnaire response rate (10 patients completed the questionnaires) and the inability to interview patients as the potential burden was deemed to be too high. A more comprehensive evaluation of the patients’ perspectives could have provided more patient-driven issues for this evaluation. In addition, only 11 community nurses returned a completed questionnaire. However, considering that there were only 14 community nurses in total, the response rate was relatively high. Furthermore, the out-of-territory perspective from clinicians also had limited representation, as this study had access to only 4 types of out-of-territory specialists (dermatology, psychiatry, orthopedics, and ophthalmology), which corresponds to the clinical specialties that were already delivering telehealth or face-to-face care to Yukon citizens by out-of-territory specialists. Finally, the clinicians who agreed to be interviewed or to participate in focus groups may have been more interested or had a more positive opinion of telehealth than the clinicians who did not participate in the study.

### Conclusions

This evaluation found that there are significant opportunities to improve and expand the Yukon Telehealth System, which has plateaued in the number of telehealth consultations since 2008. These opportunities include the expansion of services in several new specialty areas, updating telehealth equipment to streamline workflows and increase convenience and uptake, and integrating novel technologies such as telemonitoring, education tools, and online programs. This expansion would be facilitated by the current general positive perceptions of the Yukon Telehealth System by both patients and clinicians. The factors that have been historically challenging to expansion and should be considered while the system evolves include patient and clinician buy-in, workflow, access to telehealth technology, and infrastructure with regard to human resources and technology. These factors and the lessons learned from this case study can be valuable considerations for the development of new telehealth programs in remote communities and for programs that may have plateaued in use.

## Abbreviations

AA: Alcoholics Anonymous
